# Double Resection of the Lower Maxilla

**Published:** 1898-10

**Authors:** Eugene S. Talbot


### DOUBLE RESECTION OF THE LOWER MAXILLA.

BY EUGENE S. TALBOT, M.D., D.D.S.[1]

IN the *Dental Cosmos* for August, 1898, appears an article by Dr. Edward H. Angle, of St. Louis, upon the above subject, in which he claims to be the first to suggest this operation, and that it had been discussed for four years by surgeons with regard to the prognosis, hence he deems some suggestions as to technique and plans of fixation not amiss, especially since he claims, if the operation be properly performed, it may become of considerable use and importance. In those extreme types of malocclusion familiar to every one, he remarks, the proper functions of the teeth have become almost wholly impaired, as well as speech greatly interfered with, and the appearance of the patient transformed to marked deformity, constantly attracting attention and comment and being a source of humiliation to the patient, whose condition, if he be possessed of a sensitive nature, becomes truly pathetic.

The late Dr. W. W. Allport, who suggested this operation over four decades ago, consulted the late Dr. Brainard, of Chicago, admittedly then one of the most skilful American surgeons, in regard to its propriety. Sections of the inferior maxilla were to be removed at the location of the first bicuspid. The section to be cut in such a manner as to bring the incisors directly in contact with the superior incisors, and not throw the anterior part of the jaw downward, as would be the case in Dr. Angle's operation. Later, he consulted Drs. Moses Gunn and Powell, of Chicago. They were all of the opinion that such an operation could not be successful, first, owing to the impossibility (at that time, as they supposed) of holding the parts steady in place. Second, that the arteries and nerves would not unite, thus producing death of the pulps or teeth in the anterior part of the jaw; hence the operation was not performed. The first difficulty can, no doubt, be overcome by modern methods. The second requires demonstration.

The report of a case in the July *Dental Cosmos* does not indicate a very successful operation.

"At the end of the third week Dr. Blair brought Mr. K. to my (Dr. James W. Whipple, St. Louis, Mo.) office, when, to my regret, I found a very serious and unfortunate condition of affairs existing. The means adopted for holding the superior borders of the dissevered maxilla in position had proved to be entirely ineffective.

---

[1] Fellow of the Chicago Academy of Medicine.

The parts had failed to unite on either side. On the left side there was an effusion of bloody pus, which oozed into the mouth when the parts were pressed together. The right side was in a better condition, but no union of the bone had been effected. The jaw was stiff and sore, and the teeth could be separated from the upper only a short distance."

The operation was performed December 19; Dr. Whipple was still trying to adjust an appliance to hold the parts together on the 26th of the following April. If the pulps in the eight anterior teeth be alive, it is a revelation in vitality of nerve and blood-vessel tissue.

If such an operation were possible I should quite disagree with Dr. Angle as to the method of improving the appearance of this person's face. What he needs is an expression of character. The deformity is the result of arrest of development of the face and superior maxilla rather than excessive development of the lower jaw, hence I should suggest an improvement of the face generally. The deformity should be corrected by bringing forward the upper teeth (making the cuspids prominent) and the alæ of the nose, thus giving character to the face rather than increasing the deformity and further humiliating the patient.

